# The Place of Orthopedic Surgery in an Army Organization

**Published:** 1918

**Authors:** 


### WAR MEDICINE SURGERY & HYGIENE

### RESEARCH SOCIETY REPORTS

THE SIXTH SESSION OF THE AMERICAN RESEARCH SOCIETY
Hotel Continental, May 17 and 18, 1918

*Major Walter B. Cannon, Chairman of the Research Committee of the A. R. C., presided throughout the session The subject of the first meeting of the session was Military Orthopedics.*

### MILITARY ORTHOPEDICS

**The Place of Orthopedic Surgery in an Army Organization.** Major JOEL E. GOLDTHWAITE, M. R. C., Senior Consultant in Orthopedic Surgery, read a paper in which he stated that the work of the Division of Orthopedic Surgery in the medical organization of the army divides itself clearly into two parts, one concerned with preparing men for combat and the other with assisting in their recovery if wounded.

*Pre-Combat Orthopedic Work.*

*a*) This involves : —

1. Instruction in the proper use of the body in standing, walking, or other activities, so that there is the least possible waste of energy or liability to over-strain in the performance of regular duties.

2. Special training, by means of drills, orthopedic exercises, etc., to overcome bad habits of carriage or body use.

3. Instruction in the care of the feet.

4. Instruction of the stretcher bearers in the use of standardized splints for transport of the wounded.

*b*) Great numbers of men are " scrapped " from the army not because they are sick but because of symptoms which have developed from wrong usage of the body. Once the bad habits are overcome, foot weakness and back strain (the most common results of postural defects) will disappear, for such conditions represent weakness and not disease. Treatment must be carried out on a group basis, so that large numbers can be cared for simultaneously.

*Special Training Battalion.* Men with specially troublesome feet, weak backs, general bad posture, lack of endurance, etc. are sent to this battalion for training. Every minute of the time the fully erect, alert position of the body is emphasized, while every movement involved in special tasks is made from the alert or "attention" position. Four companies have been established in the battalion, with programs of increasingly difficult planning so that the schedule of the fourth company is only slightly below that required for full combat fitness. Before the final discharge is made the men must demonstrate by involuntary use of the body that the corrected habits of posture have become automatic. With the methods adopted most of the men can not only be saved for the army but between 70 o/o and 80 o/o of them can be made fit for full combat duty.

Much of the time of the representatives of the Division of Orthopedic Surgery has been occupied in supervising the shoeing of the army. If the common pronation or inward sag of the ankle can be corrected by raising the inner edge of the heel, the normal strength is regained. With markedly spread feet the use of figure-of-eight marching straps, together with the raised inner edge of the heel, gradually draws the foot together and normal tone ultimately results. For the men of the Special Training Battalion, a shop has been established where 100 pairs of shoes can be balanced in a day and in which the necessary leather foot straps can be made.

When the men are ready for combat it is important to see that the least possible injury results from wounds which may be received. To lessen the risk of careless or incompetent handling in the transport to hospital the Chief Surgeon has adopted a standardized set of splints for the army, and the instruction of the stretcher bearers and ambulance corps men in their use has become part of the work of the Orthopedic Division.

*Post-Combat Period.*

A most perfect surgical result in the Advanced Zone may become a very poor functional result unless proper supervision and direction of the case is assured to the man when he is sent to the rear. To meet the needs of these cases, special hospitals are being organized in the rear where the work will be performed partly by orthopedic surgeons and partly by general surgeons interested in orthopedics and desiring assignment to that branch. Not only will there be the usual methods for the rapid restoration of function, but curative workshops will also form part of the equipment of these hospitals. All cases of major amputation will be sent there for appropriate treatment to prevent the common contractures of the joint above the stump, and there also the temporary artificial limbs will be fitted with the idea of encouraging function at the earliest possible moment.

It is obvious that the work of the Orthopedic Division helps the men as individuals as well as saving them for the army. The preliminary training with the better habits of carriage makes for better general health, and the physical training which insists upon alertness of body also results in much greater alertness of mind. In regard to the post-combat work, a man with an artificial leg may be just as useful for office work or other inactive service for the army as a man with two normal legs, and if he is made to realize that he need not be " scrapped " but is recognised as of use his *morale* is preserved and a standard set that will be carried into civil life. The work, therefore, while primarily military, is at the same time most broadly humanitarian and represents a square deal to the man who has played squarely to the nation.

